# An evaluation of the German version of the Sensory Perception Quotient from an expert by experience perspective

**DOI:** 10.3389/fpsyg.2024.1252277

**Published:** 2024-02-29

**Authors:** Afton M. Bierlich, Carola Bloch, Timo Spyra, Christian Lanz, Christine M. Falter-Wagner, Kai Vogeley

**Affiliations:** ^1^Department of Psychiatry and Psychotherapy, LMU University Hospital Munich, LMU Munich, Munich, Germany; ^2^Department of Psychiatry and Psychotherapy, Faculty of Medicine and University Hospital Cologne, University of Cologne, Cologne, Germany; ^3^Cognitive Neuroscience, Institute of Neuroscience and Medicine (INM-3), Research Center Jülich, Jülich, Germany

**Keywords:** sensory perception quotient, autism, sensory sensitivity, participatory research, translation

## Abstract

Sensory processing is often altered in individuals with autism; thus, it is essential to develop reliable measurement tools to assess sensory perception. The Sensory Perception Quotient (SPQ) quantifies basic sensory sensitivities in adults via self-report. Adopting an expert by experience perspective, this study aimed to evaluate a German translation of the SPQ for its use in clinical and research applications, especially for autistic adults. 108 adults (*n* = 54 autistic) completed the German SPQ in an online assessment. A 92-item and a 35-item version of the German SPQ were analyzed for group differences and internal consistency. Our results show that adults with autism reported greater sensory sensitivity compared to non-autistic adults. Results further suggest good to excellent internal consistency for the 95-item and 35-item SPQ translations. This finding was supported by the correlative relationship between sensory sensitivity and autistic traits. These findings confirm the reliability of our SPQ translation, making it a suitable German assessment tool for basic sensory sensitivity in autistic adults.

## 1 Introduction

The DSM-5 (American Psychological Association, 2013) includes altered sensory processing as a sub-diagnostic component of Autism Spectrum Disorder (ASD), emphasizing the relevance for a suitable measure targeting basic sensory sensitivity.

A vast collection of research has investigated atypical sensory processing in autism, showing differentiated neural and behavioral processes (Baum et al., 2015; Robertson and Baron-Cohen, 2017; Jassim et al., 2021). Yet, the direction of these alterations remains unclear in light of the mixed findings reported across sensory processing domains (Robertson and Baron-Cohen, 2017; Hadad and Yashar, 2022). Sensory processing literature, especially in the visual domain, shows a wide range of perceptual abilities in autism. For example, behavioral findings (Happé, 1996; Bölte et al., 2007) and neural modeling approaches based on neuroimaging, perceptual, and biological studies (Park et al., 2022) show that visual illusions are processed differently by autistic individuals, while others show comparable susceptibility (e.g., Milne and Scope, 2008; for review, see Hadad and Yashar, 2022). Other works related to visuo-spatial and visuo-cognitive perception have shown differentiated processing patterns, e.g., task-specific top-down modulation (Intaitė et al., 2019) but intact visual updating abilities (Weber et al., 2021), enhanced figure-disembedding (Falter-Wagner et al., 2022), and varied visuo-temporal processing capabilities depending on the task (Falter et al., 2012; Casassus et al., 2019; Poole et al., 2022). More generally, the same mixed findings are also apparent within the touch domain, showing both hypo- and hypersensitivity in tactile detection (Robertson and Baron-Cohen, 2017). Considering the apparent, although directionally heterogeneous (hypo−/hyper-), differences in sensory perception, clinicians and researchers need broad and consistent measures to easily and reliably evaluate sensory perception.

Tavassoli et al. (2014) introduced the Sensory Perception Quotient (SPQ), a tool to assess basic sensory sensitivities in autistic adults. The SPQ is comprised of 92 items and assesses sensitivity across the five sense domains: vision, touch, smell, taste, and hearing. The statements are phrased to target one’s ability to detect or discriminate the sensation. Half of the items are worded to reflect hypo- and hyper-sensitivity, which captures the mixed findings in the context of atypical sensory profiles in autism. Responses to each item range from 0 (“strongly agree”) to 3 (“strongly disagree”), with hypo-sensitive statements reverse coded. Based on a Principal Components Analysis (PCA), Tavassoli et al. (2014) also derived a shorter 35-item version of the SPQ, that included the respective sensory domains. In a sample of adults with and without autism,^1^ Tavassoli et al. (2014) demonstrated excellent reliability of the 92-item and 35-item versions of the SPQ. Autistic adults reported greater sensory sensitivity than non-autistic adults, and autistic traits correlated strongly with sensory sensitivity.

Although there are a variety of sensory questionnaires available, some have been especially developed and validated in autistic samples (see DuBois et al. (2017) for a review). Around the same time as the SPQ’s conception, the Glasgow Sensory Quotient (GSQ; Robertson and Simmons, 2013) emerged as a useful assessment tool to evaluate sensory sensitivity in relation to autism-like traits. In addition to the common sensory domains, the GSQ additionally assesses vestibular and proprioceptive sensitivities. Its development also contrasts the SPQ as items were partly derived via qualitative analysis (Robertson and Simmons, 2013; DuBois et al., 2017), whereas the SPQ items were derived considering properties of physiological receptors belonging to each sensory domain (Tavassoli et al., 2014). Most importantly, the GSQ aligns with other measures, such as the Adolescent/Adult Sensory Profile (Brown et al., 2001) and Sensory Over-reactivity Scale (Schoen et al., 2008), as it uses affective language. Therefore, Tavassoli et al. (2014) developed the SPQ because it strips away affective and cognitive language to discriminate basic sensory sensitivity perception; thus, filling the gap in the clinical and scientific repertoire as a sensory sensitivity assessment, specifically for adults.

Reliable translations of questionnaires are vital to ensure that measures are consistent across languages. Since its development, numerous translations of the SPQ have been conducted [see the Autism Research Centre (2023) repository], including a published Dutch version (Weiland et al., 2020). Similar to the present study, Weiland et al. (2020) focused on the adaptation of the SPQ-short in relation to the Dutch AQ-short. At the time of study pre-registration, there was no other German translation of the SPQ, nor were there openly available and easily applicable German questionnaires assessing sensory sensitivity in adults. Recently, Klein et al. (2022) also report a German translation and alternative short version of the SPQ. Regarding similarities, both teams adopted the forward and backward translation approach in addition to group discussions with clinical experts to refine the final version. In addition, the current version was translated by our autistic co-author and confirmed with experts by experience. Alternative from our approach, Klein et al. (2022) used a different approach in deriving a short, translated version of the SPQ that was based on the power of the individual items for group differentiation. We aimed to derive a short version of our translation emulating the methods employed by Tavassoli et al. (2014). Notably, because the respective studies were concurrently conducted, it was not possible to comparatively assess the versions in application.

In light of the reliable psychometric properties of the SPQ (Tavassoli et al., 2014) and the lack of German questionnaires assessing sensory sensitivity at the time of pre-registration of the current study, this emphasized the relevance for clinical application in German-speaking countries. Thus, in a participatory research approach, we translated the SPQ in synergy with our coauthor and licensed translator with autism (CL). Our version has been publicly available via the Autism Research Centre (2023) since 2020 and was evaluated in a sample of adults with and without autism.

## 2 Methods

The Ethics Committee of the University Hospital of Cologne approved this study (no.: 20-1081). The preregistration is available at: osf.io/a4npv.

### 2.1 Participants

An *a priori* power analysis was conducted, assuming a fixed effect analysis of variance (ANOVA), an estimated effect size (d) of 0.28 as derived from Tavassoli et al. (2014), a power of 0.80, and an alpha of 0.05. A sample of 103 participants was deemed sufficient to detect an effect.

Participants with a confirmed diagnosis (F84.0, F84.5) according to ICD-10 (World Health Organization, 2016) were recruited through the autism outpatient clinics for adults at the University Hospital Cologne and LMU University Hospital Munich, where they were previously diagnosed.^2^ Non-autistic participants were recruited via local channels. General inclusion criteria were declaration of consent and a minimum age of 18 years.^3^

The final sample included 54 autistic (21 identified as female; 33 identified as male; age: M ± SD = 43.74 ± 12.37; verbal IQ: M ± SD = 102.24 ± 9.45), and 54 non-autistic (21 identified as female; 33 identified as male; age: M ± SD = 42.89 ± 13.04; verbal IQ: M ± SD = 94.91 ± 7.26) individuals. Additional characterizations of the sample, including reported comorbidities and reported education, are reported in the Supplementary material.

### 2.2 Translation

We utilized a simple direct translation approach that was finalized by a committee, as described by (de la Puente et al., 2001), to generate a final version of the translated questionnaire. We aimed to avoid ambiguity and to convey similar meaning of expressions in German, especially considering the subjective nature of sensory-related issues. Our translation team included trained clinicians and experts by experience, who independently translated the SPQ from English into German (CB; non-autistic) and back-translated from German to English (CL; autistic and certified translator). Comparison of the English versions through group discussion (CB, CL, CFW; non-autistic) prompted further adjustments, resulting in the final translation. Contrastingly, we did not employ a pre-test.

### 2.3 Procedure

Participants completed an online assessment of demographic data and German versions of the Sensory Perception Quotient (Tavassoli et al., 2014), the Autism-Spectrum Quotient (AQ; Baron-Cohen et al., 2001), Adult Developmental Co-ordination Disorders/Dyspraxia Checklist (ADC; Kirby et al., 2010), the Toronto Alexithymia Scale (TAS; Bagby et al., 1994), and the Wortschatztest (WST – a German vocabulary test; Schmidt and Metzler, 1992). Only the SPQ, AQ, and WST are reported in the present study. Participants were compensated 10 euros for their participation.

### 2.4 Data preprocessing

Data processing was conducted in RStudio (v2022.07.1; RStudioTeam, 2022). Items on the AQ were missing for two autistic participants and six non-autistic participants (maximally five items per person). Nine autistic participants and two non-autistic participants had missing items (maximally 14 items) on the SPQ, which underwent rounded group mean imputation.

### 2.5 Analysis

Mann–Whitney U-tests were conducted for group comparisons on non-normal data. We opted for linear models of two categorical variables (diagnostic group, gender), using sum-contrast coding, in which assumptions were inspected (homoscedasticity, no multicollinearity, normality of residuals). Although the original power analysis was conducted with an ANOVA, our sanity checks revealed comparable findings with the linear model. Cronbach’s alpha and McDonald’s omega were calculated for both translation for each group to assess reliability and comparability.

Principal Components Analysis (PCA) was conducted separately per group to investigate the latent structure of the German SPQ-92. Due to the ample statistical power necessary for factor analysis, we report the PCA and derived short version in the Supplementary material as we intended to evaluate whether the German translation would yield comparable results to Tavassoli et al. (2014).

## 3 Results

### 3.1 Descriptive statistics

The groups were matched for age (*U* = 1,408, *p* = 0.763) and gender identity but differed in verbal IQ performance (*U* = 745, *p* < 0.001). Groups differed in autistic traits (*U* = 3.5, p < 0.001), with higher AQ scores for autistic (M ± SD = 39.96 ± 5.35) compared with non-autistic (M ± SD = 16.91 ± 5.56) participants. Correlation analysis demonstrated no significant correlation between verbal IQ and any SPQ in either group (Table 1).

**Table 1:** Correlation matrix of sensory sensitivity, autistic traits, and verbal IQ.

	SPQ-92	SPQ-35	AQ
*Autistic group*			
SPQ-92	–		
SPQ-35	**0.96**	–	
AQ	−0.37	−0.41	–
WST	−0.08	−0.05	0.14
*Non-autistic group*			
SPQ-92	–		
SPQ-35	**0.88**	–	
AQ	0.00	−0.09	–
WST	−0.10	−0.18	−0.05

Pearson’s *r* correlation coefficients. Bolded values remained significant (*p* < 0.05) after Bonferroni correction.

### 3.2 Reliability and comparability

Internal consistency was good to excellent for our SPQ-92 (autistic: α = 0.93, ω*_h_* = 0.48; non-autistic: α = 0.83, ω*_h_* = 0.71) and SPQ-35 (autistic: α = 0.94, ω*_h_* = 0.64; non-autistic: α = 0.84, ω*_h_* = 0.37). In line with Tavassoli et al. (2014), the SPQ-92 strongly correlated with the SPQ-35 for both groups (Table 1). Additionally, an item per item comparison between groups is presented in the Supplementary material.

### 3.3 SPQ group comparisons

**Figure 1:**
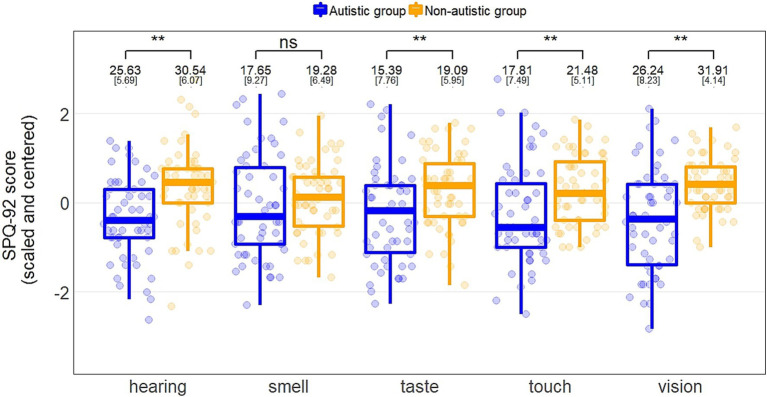
Boxplots of scaled and centered scores on each SPQ-92 subscale for individuals with and without autism. Group comparison of subscales from independent sample *t*-tests (hearing, touch) and Welch’s *t*-tests where variance was heterogeneous (vision, smell, taste), including means and standard deviations in raw values.

Linear regressions, with group and gender identity as predictors, were conducted for the SPQ-92 and the SPQ-35 (Table 2). For all models, participants with autism reported significantly greater sensory sensitivity than non-autistic participants, and females reported significantly greater sensitivity than males. Figure 1 depicts the group effects between the SPQ-92 subscales. A supplementary analysis accounted for group differences on verbal IQ, whereby the group and gender effects remained robust (see Supplementary material).

**Table 2:** Group comparison of the translated SPQ-92 and SPQ-35.

		SPQ-92	SPQ-35
Autistic group		102.72 (32.14)	42.94 (19.86)
Non-autistic group		122.30 (19.77)	53.39 (11.55)
Group difference?	*Β*	10.89***	6.13***
	*95% CI*	[6.21, 15.57]	[3.32, 8.93]
Gender difference?	*Β*	−11.48***	−6.95***
	*95% CI*	[−16.16, −6.80]	[−9.76, −4.15]

Means and standard deviations for the SPQ-92 and SPQ-35 are reported for each group. The beta coefficient (*B*) and 95% Confidence Intervals (*CI*) are reported as main effects, with significance levels of 0.05 indicated (**p < 0.01, ***p < 0.001).

### 3.4 AQ and SPQ

**Figure 2:**
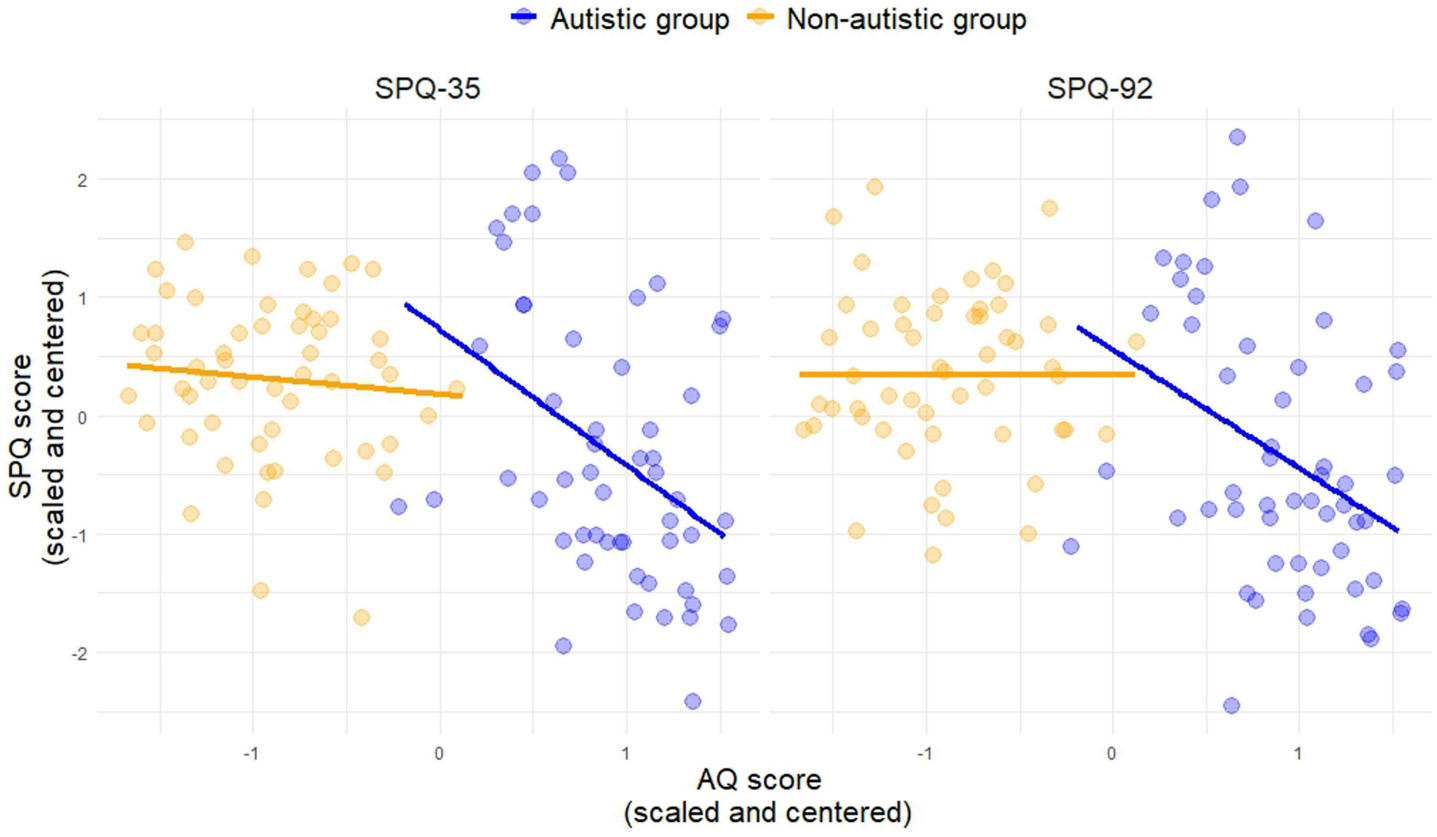
The panels depict data from each SPQ version and the AQ for both groups.

The correlation between autistic traits (AQ) and sensory sensitivity was significant in the group with autism but did not survive Bonferroni correction (Table 1; Figure 2).

## 4 Discussion

This pre-registered evaluation study of a German translation of the SPQ (Tavassoli et al., 2014) demonstrated good psychometric properties of the SPQ-92 and SPQ-35 suggesting efficacy for their use in clinical and research settings.

Consistent with previous literature (Tavassoli et al., 2014; Klein et al., 2022), autistic adults reported greater sensory sensitivity compared to non-autistic adults. This finding was strengthened by the significant relationship between sensory sensitivity (SPQ) and autistic traits (AQ) in the group of individuals with autism (SPQ-92: *p* = 0.006; SPQ-35: *p* = 0.002), although these effects did not survive multiple comparison correction.^4^ Furthermore, individuals who identified as female reported greater sensory sensitivity than those identifying as male. Although there were more participants who identified as male in the sample, this was comparable across diagnostic groups. This is in line with previous findings reporting gender differences (Tavassoli et al., 2014; Klein et al., 2022), especially in the females with autism (Lai et al., 2011; Osório et al., 2021), and may hint at a gender-specific sensory profile in autism.

Notably, the present investigation focuses on the use of the current German SPQ in autistic and non-autistic individuals, given that sensory sensitivities are part of the diagnostic profile. However, the present sample was comprised of individuals with autism who had average verbal IQ performance (M ± SD = 94.91 ± 7.26). As such, our findings may not extend to sub-populations of autistic individuals, such as those who are non-verbal or with intellectual disabilities or giftedness. Moreover, it should be noted that sensory sensitivities are also relevant for other clinical populations, such as ADHD (e.g., Bijlenga et al., 2017; Kamath et al., 2020), borderline personality disorder (Rosenthal et al., 2011; Amerio et al., 2023), schizophrenia (Haigh et al., 2017; Robertson and Baron-Cohen, 2017), traumatic brain injuries (Callahan and Lim, 2018), and chronic pain (Schrepf et al., 2018). Future research should apply the SPQ and its translations to other clinical populations to determine its efficacy in the respective populations.

Providing a reliable short version of the German SPQ is certainly valuable for clinical and research application. However, the small sample size of the present study is a clear limitation for a PCA and, thus, calls for replication and confirmation in future work. The present study intended to follow the original factor analysis approach employed by Tavassoli et al. (2014) to assess whether the German translation would yield similar results. Considering the limits of the sample size, the PCA and explanation of the derived short SPQ is reported in the Supplementary material.

Furthermore, the use of self-report to measure sensory sensitivity should be critically considered, given that self-report tools are subjective and may not reflect one’s actual state. However, self-report assessments are often used in clinical practice as they can be readily applied and provide clinicians and researchers with a general overview when applied correctly. Similarly, the WST was used to assess verbal IQ to compare the respective autistic and non-autistic samples. It is still often used in clinical practice, as it is quite short and easy to administer; however, future research may consider using a more recent verbal IQ assessment for such purposes.

Notably, questionnaires require translations for use across languages. Objective measures, like psychophysical methods, offer a robust approach for assessing sensory perception as they are language invariant. Pairing such subjective and objective approaches may enhance the reliability of translations across languages. Moreover, deriving reliable questionnaire translations, as we aimed to accomplish with the German SPQ, are necessary to allow for complementary assessments of broad sensory profiles as they relate to the underlying neural mechanisms of sensory perception. For example, neural modeling approaches based on neuroimaging, perceptual, and neurobiological studies have reported reduced top-down modulation and a neuronal inhibition/excitation imbalance in autistic (Park et al., 2022) and schizophrenic (Zhu et al., 2023) individuals during visual illusion processing. However, other perceptual paradigms demonstrate distinct neural and behavioral sensory processing profiles for autistic individuals and schizophrenic individuals (Robertson and Baron-Cohen, 2017). Thus, the range of neural mechanisms underlying sensory perception in autism and other psychiatric conditions warrants complementary assessment with consistent measures across languages.

Importantly, the present study fills a gap in the clinical repertoire of German assessments. Considering that two German translations of the SPQ exist, future investigations should compare them using the same sample, as well as the profiles on an item per item basis, as the studies were concurrently conducted. Furthermore, to our knowledge, there were no other validated German sensory sensitivity assessments available at the time of study pre-registration; therefore, it was not possible to evaluate the concurrent validity of our translation. This highlights the importance in providing a reliable clinical measure of sensory sensitivity for use in German populations.

## 5 Conclusion

The present study aimed to evaluate a German SPQ from an expert by experience approach. Given that our translation of the SPQ is already in use, a validation was imperative for open and reproducible science. Our findings confirm validity of our translation for the SPQ-92 and SPQ-35, converging with the original findings (Tavassoli et al., 2014) that the SPQ-35 sufficiently assesses sensory sensitivity for use in clinical and research settings. Future studies should run comparative analyses to investigate concurrent validity and comprehensibility from an expert by experience perspective.

## Data availability statement

Upon request and after anonymization, the raw data supporting the conclusions of this article will be made available by the authors, without undue reservation.

## Ethics statement

The studies involving humans were approved by the Ethics Committee of University Hospital of Cologne. The studies were conducted in accordance with the local legislation and institutional requirements. The participants provided their written informed consent to participate in this study.

## Author contributions

AB: methodology, formal analysis, visualization, writing – original draft, and writing – review & editing. CB: conceptualization, project administration, resources, formal analysis, and writing – review & editing. TS: project administration, investigation, methodology, and writing – review & editing. CL: resources and writing – review & editing. CF-W: conceptualization, project administration, supervision, resources, funding acquisition, and writing – review & editing. KV: conceptualization, project administration, supervision, funding acquisition, and writing – review & editing. All authors contributed to the article and approved the submitted version.

## Footnotes

^1^We acknowledge the different preferences concerning the choice of language used to describe the autism community (i.e., person-first vs. identity-first), which widely varies across languages and cultures (Kenny et al., 2016; Lei et al., 2021; Tepest, 2021; Buijsman et al., 2022; Bury et al., 2022; Keating et al., 2023). We alternate between ‘autistic adults’ and ‘adults with autism’ throughout the report.

^2^The study was advertised within the outpatient clinic for autistic adults at the LMU University Hospital Munich, but all data were collected online through the University Hospital of Cologne.

^3^In line with the inclusion criteria of Tavassoli et al. (2014), a supplementary analysis including a smaller sample with an Autism-Spectrum Quotient restriction is included in the supplementary material.

^4^Although, see the shortened SPQ-13 derived from the present PCA in the Supplementary Material, as this relationship with autistic traits remained robust after multiple comparison correction.
