# Corrigendum: An evaluation of the German version of the Sensory Perception Quotient from an expert by experience perspective

**DOI:** 10.3389/fpsyg.2025.1565601

**Published:** 2025-03-25

**Authors:** Afton M. Bierlich, Carola Bloch, Timo Spyra, Christian Lanz, Christine M. Falter-Wagner, Kai Vogeley

**Affiliations:** ^1^Department of Psychiatry and Psychotherapy, LMU University Hospital Munich, LMU Munich, Munich, Germany; ^2^Department of Psychiatry and Psychotherapy, Faculty of Medicine and University Hospital Cologne, University of Cologne, Cologne, Germany; ^3^Cognitive Neuroscience, Institute of Neuroscience and Medicine (INM-3), Research Center Jülich, Jülich, Germany

**Keywords:** sensory perception quotient, autism, sensory sensitivity, participatory research, translation

In the published article, there was an error in Supplementary Tables 6 and 9. There was a typographical error in the coding of items 88 and 89 in Supplementary Tables 6 and 9. Italicization indicates items that are reverse coded. In the original Supplementary Tables, item 88 was italicized, suggesting it was reverse coded, while item 89 was not italicized, indicating normal coding. The corrected tables show that item 88 should not be italicized, indicating it is normally coded, and item 89 should be italicized, indicating it is reverse coded. This typo was present only in the Supplementary Tables and did not affect the data processing and analysis, which were correct. The correct material statement has been published in the original article.

The authors apologize for this error and state that this does not change the scientific conclusions of the article in any way. The original article has been updated.

