# Supplementary material for: An evaluation of the German version of the Sensory Perception Quotient from an expert by experience perspective
Source: Front Psychol. 2024 Feb 29;15:1252277. doi: 10.3389/fpsyg.2024.1252277 (PMC10937587; doi:10.3389/fpsyg.2024.1252277)
Supplement: Supplementary file 1 [file Data_Sheet_1.docx]

Supplementary Material

An Evaluation of the German Version of the Sensory Perception Quotient (SPQ) from an Expert by Experience Perspective

Afton M. Bierlich ^†*^, Carola Bloch^†*^, Timo Spyra, Christian Lanz, Christine M. Falter-Wagner^††^ and Kai Vogeley^††^

^†^ **These authors share first authorship.**

^††^ **These authors share last authorship.**

*** Correspondence: ss**

Afton Bierlich: [afton.nelson@med.uni-muenchen.de](mailto:afton.nelson@med.uni-muenchen.de).

Carola Bloch: [carola.bloch@med.uni-muenchen.de](mailto:carola.bloch@med.uni-muenchen.de).

# Demographics

Sample demographics, including the education, employment, reported psychiatric and neurological conditions, as well as medication in-take, are reported. Race and ethnicity were not recorded during data collection.

Supplementary Table 1. Reported current employment status of the sample. 4.62% reported being students, and 10.18% reported being students with employment (a student job*, part-time, full-time). 10.19% reported part-time employment, and 52.78% reported full-time employment. 0.93% reported being a stay-at-home spouse. 0.93% reported being self-employed. 12.96% were unemployed, unable to work due to psychological or physical ailments, or were on long-term sick leave. 0.93% reported a voluntary social year. 4.62% did not specify. 1.85% were unclear. A student job refers to a part-time employment earning 450 Euros or less, as is common for students in Germany.

| **Current Employment** | **Frequency** |
| --- | --- |
| Student | 5 |
| Student with a student job | 4 |
| Student with irregular employment | 1 |
| Student with part-time employment | 3 |
| Student with part-time employment and a student job | 1 |
| Student with full-time employment | 1 |
| PhD | 1 |
| Part-time employment | 11 |
| Full-time employment | 57 |
| Employed by organization for people with disabilities | 2 |
| Long-term sick leave | 1 |
| Stay at home wife/husband | 1 |
| Self-employed | 1 |
| Retired, unable to work due to psychological reasons | 1 |
| Unable to work due to psychological and physical reasons | 1 |
| Unable to work due to psychological reasons | 5 |
| Applying for retirement, unemployed, disabled | 1 |
| Federal Voluntary Service, Voluntary Social Year | 1 |
| Unemployed | 5 |
| N/A | 5 |

**Supplementary Table 2.** Reported education of the sample. Upon visual inspection of the responses, only the highest achievement reported was included in the table when one or more educational achievements were reported. 5.56% reported being currently enrolled in an apprenticeship training. 4.62% reported finishing an apprenticeship in a company and undertaking enrollment in a second apprenticeship training. 0.93% reported finishing advanced Realschule. 14.81% completed an internship in a company, 11.11% completed an apprenticeship in a technical school, and 1.85% completed an internship in both a company and a technical school. 11.11% completed additional training for their internship at a technical school, and 8.33% completed a bachelor’s degree at a technical school. Further education (e.g., a master’s or Diplom degree) from a technical school was obtained by 13.89% of participants. 15.74% completed a bachelor’s degree from a university, and 2.78% completed a PhD. 3.70% reported no degree completion or ongoing apprenticeship. 4.62% did not specify. 0.93% were unclear. Some terminology reported are specific to the German educational system and do not have an exact counterpart or translation.

| **Highest Education** | **Frequency** |
| --- | --- |
| Currently in apprenticeship training | 6 |
| Apprenticeship in a company + currently in apprenticeship training | 5 |
| Advanced Realschule degree | 1 |
| Apprenticeship in a company | 16 |
| Apprenticeship in a technical school | 12 |
| Apprenticeship in a company + Apprenticeship from a technical school | 2 |
| Apprenticeship in a technical school (continued training) | 12 |
| Bachelor’s degree from a technical school | 9 |
| Degree from a technical school (Masters, Diplom) | 15 |
| University degree (Masters, Diplom) | 17 |
| PhD | 3 |
| No degree or ongoing apprenticeship | 4 |
| N/A | 5 |
| Unclear | 1 |

Supplementary Table 3. Reported psychiatric conditions or comorbid psychiatric conditions with autism. Considering the online nature of this study, *self-reported* comorbid conditions and medication in-take could not be confirmed. Reported psychiatric conditions were categorized through visual inspection. Examples of ‘Other Reported Diagnoses’ mentioned include ADHD, social phobias, anxiety disorders, personality disorders, amongst others. Given that recruitment occurred through clinical research databases, a diagnosis of Autism Spectrum Disorder was considered confirmed. Other reported psychiatric diagnoses could not be confirmed due to the online nature of the study. In most cases, participants not explicitly report F-diagnoses; thus, we cannot confirm the actuality of the reported diagnoses. We acknowledge this as a limitation in the study design.

| **Non-autistic group** | **Frequency** |
| --- | --- |
| Psycho-somatic symptoms | 1 |
| Mild depressive symptoms | 1 |
| Anxiety and panic disorders | 2 |
| **Autistic group** |  |
| Autism Spectrum Disorder (F84.5 or F84.0) | 19 |
| Autism Spectrum Disorder (F84.5 or F84.0) + Depression | 12 |
| Autism Spectrum Disorder (F84.5 or F84.0) + Depression + Other Reported Diagnoses | 13 |
| Autism Spectrum Disorder (F84.5 or F84.0) + Other Reported Diagnoses | 8 |

Supplementary Table 4. Reported neurological conditions. Because neurological conditions are less common, only the number of individuals who reported a neurological are reported in order to maintain the anonymity of our participants. Examples include stroke, migraines, and spasticity, as well as other rare neurological conditions. Reported neurological diagnoses could not be confirmed due to the online nature of the study. They are reported rather than excluded, given that participants were only asked to complete online questionnaires at their own willingness. No extreme visual stimuli or sensitive methodologies were used.

|  | **Frequency** |
| --- | --- |
| **Non-autistic group** | 2 |
| **Autistic group** | 7 |

Supplementary Table 5. Reported current medication in-take. Only the number of participants who reported current medication in-take are reported. Reported medication in-take could not be specified or confirmed due to the online nature of the study. They are reported rather than excluded, given that medication in-take is ubiquitous in clinical samples.

|  | **Frequency** |
| --- | --- |
| **Non-autistic group** | 2 |
| **Autistic group** | 20 |

# Item per item comparison of the translated SPQ

Supplementary Table 6. Itemized group comparison including the mean and standard deviation of the translated SPQ-92.

| **Item** |  | **Autistic**  **group** | **Non-autistic**  **group** | **Domain** |
| --- | --- | --- | --- | --- |
|  |  | Mean ± SD | Mean ± SD |  |
| 1 ^b^ | Ich würde es wahrnehmen, wenn jemand 5 Körner Salz in mein Glas Wasser zugeben würde. | 1.3 ± 1.08 | 1.67 ± 1.01 | Taste-salty |
| 2 ^a^ | Ich könnte verschiedene Personen anhand ihres Geruches unterscheiden. | 1.52 ± 1.02 | 1.2 ± 0.88 | Smell-social |
| 3 | Ich würde es nicht wahrnehmen, wenn jemand einen Löffel Zucker in meinen Tee zugeben würde. | 0.37 ± 0.85 | 0.98 ± 1.11 | *Taste-sweet* |
| 4 | Ich hätte keine Angst davor mir wehzutun, wenn ich bei hoher Geschwindigkeit von meinem Fahrrad fallen würde. | 0.52 ± 0.95 | 0.78 ± 0.88 | *Touch-pain* |
| 5 | Ich würde die Bewegung der Blätter eines rotierenden Ventilators nicht wahrnehmen sogar, wenn diese sich mit Minimalgeschwindigkeit drehen. | 0.43 ± 0.74 | 0.54 ± 0.91 | *Vision-motion* |
| 6 | Der Klang eines Klaviers und einer Geige, welche die gleiche Note spielen, erscheinen mir sehr ähnlich. | 2.5 ± 0.77 | 1.74 ± 1.07 | Hearing-complexity |
| 7 ^a^ | Ich könnte alleine anhand des Geruches erkennen, ob eine Erdbeere reif ist. | 1.41 ± 1.17 | 1.63 ± 0.92 | Smell-food |
| 8 | Ich könnte Milchschokolade und dunkle Schokolade alleine am Geschmack unterscheiden. | 0.3 ± 0.63 | 0.33 ± 0.61 | Taste-sweet |
| 9 | Ich kann heiße Duschen nicht vertragen (über 40°C / 105°F). | 1.39 ± 1.22 | 1.7 ± 1.16 | Touch-pain |
| 10 | Ich bräuchte keine Betäubung, um mit einer Zahnbehandlung, wie einer Zahnfüllung, klarzukommen. | 0.93 ± 1.06 | 1.04 ± 1.05 | *Touch-pain* |
| 11 | Ich müsste 10 Minuten warten, bis ein heißes Getränk abgekühlt ist, um es runterzuschlucken, ansonsten wäre es zu heiß für mich. | 1.28 ± 1.04 | 1.59 ± 0.96 | Touch-temperature |
| 12 ^a, b^ | Ich könnte visuell Änderungen in der Helligkeit einer dimmbaren Lampe erkennen, jedes Mal wenn der Dimmer um eine Stufe bewegt wird. | 0.65 ± 0.85 | 1.13 ± 0.83 | Vision-brightness |
| 13 | Ich könnte große Objekte, wie geparkte Autos, in einer dunklen Nacht nicht klar erkennen. | 0.93 ± 0.91 | 0.83 ± 0.8 | *Vision-brightness* |
| 14 ^a, b^ | Ich würde es wahrnehmen, wenn jemand 5 Tropfen Zitronensaft in mein Glas Wasser zugeben würde. | 0.57 ± 0.77 | 0.85 ± 0.9 | Taste-sour |
| 15 | Ich wäre die letzte Person, die bemerkt wenn etwas brennt. | 0.44 ± 0.72 | 0.54 ± 0.69 | *Smell-danger* |
| 16 | Ich könnte die Vibrationen lauter Musik nicht fühlen, wenn ich neben dem Lautsprecher sitzen würde (z.B. bei einem Konzert). | 0.15 ± 0.49 | 0.43 ± 0.79 | *Touch-vibration* |
| 17 ^b^ | Ich könnte kleine Lautstärkenveränderungen von Musik nicht als Unterschied der Vibration auf meiner Haut erkennen. | 1.43 ± 1.06 | 1.35 ± 0.87 | *Touch-vibration* |
| 18 | Ich kann den Fernseher nicht hören, wenn er sehr leise ist, auch wenn andere Leute es können. | 0.54 ± 0.93 | 0.8 ± 0.9 | *Hearing-loudness* |
| 19 ^a^ | Ich könnte es hören, wenn in einer ruhigen Straße ein Blatt vom Wind bewegt wird. | 1.22 ± 1.13 | 1.76 ± 1.01 | Hearing-loudness |
| 20 | Ich könnte nicht den Unterschied zwischen zwei Stücken dunkler Schokolade schmecken. | 1.17 ± 1.08 | 1.65 ± 0.97 | *Taste-sweet* |
| 21 ^a^ | Ich könnte den Unterschied zwischen zwei verschiedenen Marken salziger Kartoffelchips herausschmecken. | 1.11 ± 1.11 | 1.31 ± 0.91 | Taste-salty |
| 22 | Wenn Leute sprechen, scheinen die Wörter miteinander zu verschmelzen. | 1.35 ± 1.05 | 1.04 ± 0.85 | *Hearing-complexity* |
| 23 | Ich kann leuchtende Farben nur für kurze Zeit betrachten. | 1.46 ± 1.19 | 2.2 ± 0.79 | Vision-color |
| 24 | Ich würde sehr leicht das Gleichgewicht verlieren, wenn ich mit geschlossenen Augen auf einem Bein stehen würde. | 1.19 ± 1.05 | 1.78 ± 0.92 | Hearing-vestibular |
| 25 | Ich könnte einen Grill aus 20 Metern Entfernung nicht riechen. | 0.61 ± 0.96 | 0.61 ± 0.76 | *Smell-food* |
| 26 | Ich kann mich nicht rundherum drehen, ohne umzufallen. | 0.89 ± 1 | 0.54 ± 0.84 | *Hearing-vestibular* |
| 27 | Ich würde einen Temperaturunterschied von 10 Grad nicht bemerken. | 0.31 ± 0.67 | 0.31 ± 0.54 | *Touch-temperature* |
| 28 | Ich kann Tee/Kaffee schwarz trinken, ohne Milch oder Zucker zuzufügen. | 1.83 ± 1.21 | 1.85 ± 1.17 | *Taste-bitter* |
| 29 | Ich kann bei Musik den Bass nicht heraushören. | 0.31 ± 0.54 | 0.72 ± 0.92 | *Hearing-frequency* |
| 30 ^b^ | Ich könnte den Unterschied zwischen frisch geschnittenem Gras und ungeschnittenem Gras riechen. | 0.61 ± 0.92 | 0.7 ± 0.9 | Smell-neutral |
| 31 ^a^ | Ich könnte das Etikett auf der Rückseite meines Oberteils nicht spüren, auch wenn ich darauf achten würde. | 0.72 ± 0.98 | 0.83 ± 0.82 | *Touch-pressure* |
| 32 ^a, b^ | Ich kann das Summen von Elektrizität in der Wand hören. | 1.83 ± 1.08 | 2.39 ± 0.86 | Hearing-frequency |
| 33 ^a, b^ | Ich kann das Flackern eines Computerbildschirmes erkennen, auch wenn das Gerät ordnungsgemäß funktioniert. | 1.37 ± 1 | 2.37 ± 0.76 | Vision-motion |
| 34 | Ich könnte nicht sagen, ob Milch verdorben ist, indem ich einfach daran rieche. | 0.63 ± 0.88 | 0.72 ± 0.74 | *Smell-food* |
| 35 ^a^ | Ich könnte kleine Veränderungen (z.B. 1 Grad Celsius) in der Temperatur des Wetters erkennen. | 2.02 ± 0.92 | 2.44 ± 0.66 | Touch-temperature |
| 36 ^a^ | Ich würde einen millimetergroßen Schnitt in meiner Haut spüren. | 0.96 ± 1.1 | 1.22 ± 0.95 | Touch-pain |
| 37 ^b^ | Ich könnte die Bewegung einzelner Blätter eines rotierenden Ventilators sehen, selbst bei Maximalgeschwindigkeit. | 1.69 ± 0.95 | 2.28 ± 0.83 | Vision-motion |
| 38 ^a^ | Ich könnte mit geschlossenen Augen den Gewichtsunterschied von zwei Münzen erkennen, die auf meinen Handflächen liegen. | 1.56 ± 0.86 | 1.72 ± 0.79 | Touch-pressure |
| 39 | Mir würde auf einem Karussell selbst bei hoher Geschwindigkeit nicht schwindelig werden. | 0.69 ± 0.93 | 1.22 ± 1.13 | *Hearing-vestibular* |
| 40 | Ich kann geschrieben Wörter auf einer Seite nicht sehen, die andere Leute sehen können. | 0.33 ± 0.64 | 0.67 ± 0.67 | *Vision-acuity* |
| 41 | Ich könnte zwei Orangen ausschließlich am Geschmack unterscheiden. | 1.44 ± 1.04 | 1.65 ± 0.76 | Taste-sour |
| 42 ^a^ | Ich könnte eine bekannte Person nicht anhand ihres Geruches von einer fremden Person unterscheiden. | 1.31 ± 1.08 | 1.09 ± 0.81 | *Smell-social* |
| 43 ^a^ | Ich könnte nicht nur anhand des Geruches erkennen, ob ein Brot altbacken ist. | 1.5 ± 1.04 | 1.59 ± 0.84 | *Smell-food* |
| 44 | Ich kann nicht nur anhand des Geruches sagen, ob meine Kleidung sauber oder schmutzig ist. | 1.56 ± 1.22 | 1.39 ± 1 | *Smell-neutral* |
| 45 ^a^ | Ich könnte das Geräusch eines Staubsaugers in jedem anderen Raum eines zweistöckigen Gebäudes erkennen. | 1 ± 1.06 | 1.19 ± 0.87 | Hearing-frequency |
| 46 | Ich könnte nicht den Unterschied zwischen geradem und ungeradem Grund wahrnehmen, wenn ich auf dem Rücksitz eines Autos sitze und darüber fahre. | 0.83 ± 1.06 | 0.78 ± 0.69 | *Touch-vibration* |
| 47 | Ich könnte eine Tasse kochendes Wasser trinken, sofort nachdem es aus dem Wasserkocher gegossen wurde. | 0.24 ± 0.7 | 0.15 ± 0.49 | *Touch-pain* |
| 48 | Ich könnte nicht den Unterschied zwischen zwei grünen Äpfeln allein anhand ihrer Farbe wahrnehmen. | 1.17 ± 1.09 | 1.2 ± 0.92 | *Vision-color* |
| 49 ^b^ | Ich könnte ein altes und ein neues Buch anhand ihrer Gerüche unterscheiden. | 0.54 ± 0.99 | 1.06 ± 0.94 | Smell-neutral |
| 50 | Ich könnte ein Straßenschild aus 30 Metern Entfernung lesen. | 1.37 ± 1.1 | 1.3 ± 0.96 | Vision-acuity |
| 51 | Ich kann nicht sagen, ob Autos, die auf der Straße an mir vorbeifahren, mit unterschiedlicher Geschwindigkeit fahren. | 0.98 ± 1.02 | 1 ± 0.8 | *Vision-motion* |
| 52 ^b^ | Ich könnte es wahrnehmen, wenn jemand 5 Körner Zucker in mein Glas Wasser zugeben würde. | 1.61 ± 1.05 | 1.98 ± 0.98 | Taste-sweet |
| 53 | Ich hätte Schwierigkeiten damit, ein einzelnes Blatt klar zu sehen, sogar auf einem nahestehenden Baum. | 2.2 ± 0.9 | 2.26 ± 0.81 | Vision-acuity |
| 54 | Ich würde es nicht schmecken, wenn jemand einen ganzen Teelöffel Salz in mein Glas Wasser zugeben würde. | 0.15 ± 0.53 | 0.43 ± 0.9 | *Taste-salty* |
| 55 ^a^ | Ich könnte den elastischen Bund meiner Socken spüren, wenn ich stehen bleiben und darauf achten würde. | 0.8 ± 0.92 | 1.43 ± 0.81 | Touch-pressure |
| 56 | Ich kann nicht den Unterschied zwischen reifen und unreifen Früchten schmecken. | 0.48 ± 0.93 | 0.7 ± 0.9 | *Taste-sweet* |
| 57 | Ich könnte für fünfzehn Sekunden auf einem Bein stehen ohne zu wackeln. | 1.33 ± 1.12 | 1.98 ± 1 | *Hearing-vestibular* |
| 58 ^a^ | Ich könnte den Unterschied zwischen augenscheinlich gleichen Süßigkeiten herausschmecken. | 1.3 ± 0.92 | 1.44 ± 0.82 | Taste-sweet |
| 59 ^a^ | Ich nehme das Gewicht und den Druck eines Hutes auf meinem Kopf wahr. | 0.57 ± 0.81 | 0.96 ± 0.91 | Touch-pressure |
| 60 ^a^ | Ich würde die Berührung eines einzelnen Haares auf meinem Handrücken spüren. | 0.69 ± 0.95 | 1.22 ± 0.92 | Touch-pressure |
| 61 ^a^ | Wenn ich entlanggehe, würde ich selbst mit geschlossenen Augen die Vibrationen eines vorbeifahrenden Lasters spüren. | 0.65 ± 0.93 | 0.67 ± 0.73 | Touch-vibration |
| 62 ^a^ | Ich könnte den kleinsten Gasaustritt von überall im Haus riechen. | 1.56 ± 1 | 1.89 ± 0.74 | Smell-danger |
| 63 ^a^ | Ich würde nicht anhand des Geruches erkennen, falls jemand das Parfum gewechselt hätte. | 0.89 ± 0.95 | 0.98 ± 0.81 | *Smell-social* |
| 64 | Ich könnte sagen, wenn ein Fahrstuhl sich in Bewegung setzen würde. | 0.31 ± 0.67 | 0.41 ± 0.53 | Hearing-vestibular |
| 65 | Ich kann sehr leicht Hundepfeifen im Park hören. | 1.7 ± 1.09 | 1.57 ± 1.02 | Hearing-frequency |
| 66 | Ich würde nicht den Unterschied zwischen verschiedenen Sorten von Salatblättern schmecken. | 0.96 ± 0.82 | 1.37 ± 0.92 | *Taste-bitter* |
| 67 | Ich könnte nicht schmecken, falls in meinem Wasserglas zwei Scheiben Zitrone wären, wenn ich es mit geschlossenen Augen trinken würde. | 0.41 ± 0.74 | 0.48 ± 0.69 | *Taste-sour* |
| 68 ^a^ | Ich kann bei hellem Sonnenschein nicht ohne Sonnenbrille rausgehen. | 1.57 ± 1.21 | 2.07 ± 0.89 | Vision-brightness |
| 69 | Ich könnte Kleingedrucktes, wie eine Seriennummer auf der Rückseite einer DVD, aus 3 Metern Entfernung lesen. | 2.52 ± 0.75 | 2.65 ± 0.76 | Vision-acuity |
| 70 | Ich bekomme leicht Reiseübelkeit (z.B. im Auto oder Seekrankheit) | 1.96 ± 1.18 | 2.06 ± 1.05 | Vision-motion |
| 71 ^a^ | Ich könnte den Temperaturunterschied einer Tasse frischen Kaffees spüren, wenn dieser eine Minute herumstand. | 1.46 ± 1.06 | 1.67 ± 0.87 | Touch-temperature |
| 72 | Ich kann sehr tiefe Töne, wie tiefe Stimmen, nicht hören. | 0.41 ± 0.74 | 0.54 ± 0.72 | *Hearing-frequency* |
| 73 ^a, b^ | Ich würde es als Erste/r hören, wenn eine Fliege im Raum ist. | 1.04 ± 0.93 | 1.7 ± 0.74 | Heairng-loudness |
| 74 ^a^ | Wenn ich einen Stapel blauer Pullover im Laden sehe, die identisch sein sollen, könnte ich Unterschiede zwischen ihnen erkennen. | 1.63 ± 1.14 | 1.74 ± 0.97 | Vision-color |
| 75 ^a^ | Ich würde einen neuen Geruch in meinem Zuhause nicht direkt vor allen anderen bemerken. | 0.69 ± 0.86 | 1.06 ± 0.79 | *Smell-neutral* |
| 76 | Ich besitze absolutes Gehör: z.B. könnte ich eine Musiknote ohne Hilfe wiederholen. | 2.19 ± 1.05 | 2.57 ± 0.63 | Hearing-complexity |
| 77 | Ich könnte ohne irgendwelche Probleme in eine Zitrone beißen. | 1.22 ± 1.11 | 1.17 ± 0.97 | *Taste-sour* |
| 78 | Ich bräuchte im Winter keinen Mantel zu tragen, selbst wenn es null Grad draußen ist. | 0.63 ± 1.05 | 0.37 ± 0.62 | *Touch-temperature* |
| 79 | Ich könnte nicht die Farbe eines Pullovers im Laden an die Farbe meiner Hose zuhause anpassen. | 1.37 ± 1.05 | 1.02 ± 0.96 | *Vision-color* |
| 80 | Ich würde nicht jede einzelne Note hören, wenn ich Musik höre. | 1.61 ± 1.16 | 2.06 ± 1.05 | *Hearing-frequency* |
| 81 ^a, b^ | Ich könnte den Unterschied zwischen den meisten Männern und Frauen riechen. | 1.72 ± 1 | 1.52 ± 0.79 | Smell-social |
| 82 | Ich entscheide mich dazu, gedeckte Farben zu tragen. | 1.13 ± 1.1 | 1.76 ± 0.85 | Vision-brightness |
| 83 | Ich höre Musik bei minimaler Lautstärke. | 1.78 ± 0.92 | 2.22 ± 0.74 | Hearing-loudness |
| 84 ^a^ | Ich könnte jede Note eines Akkords hören, auch wenn es 10 Noten wären. | 2.28 ± 0.9 | 2.72 ± 0.6 | Hearing-complexity |
| 85 ^a^ | Ich schließe Vorhänge, um helles Licht zu vermeiden. | 1.26 ± 1.12 | 2.07 ± 0.84 | Vision-acuity |
| 86 | Ich könnte nicht den Unterschied in einem Ton hören, wenn das gleiche Instrument die gleiche Note zu unterschiedlichen Zeiten spielt. | 1.46 ± 1.13 | 1.59 ± 1.06 | *Hearing-complexity* |
| 87 ^a, b^ | Ich könnte zwei verschiedene Sorten Kaffee anhand ihres Geruches unterscheiden, sogar mit geschlossenen Augen. | 1.41 ± 1.14 | 1.69 ± 0.82 | Smell-food |
| 88 ^a, b^ | Ich kann in den meisten Umgebungen Staubpartikel in der Luft sehen. | 1.57 ± 1.04 | 2.17 ± 0.84 | Vision-acuity |
| 89 ^a^ | Ich könnte den Unterschied zweier Tomatensoßen mit unterschiedlichem Salzgehalt nicht herausschmecken. | 1.17 ± 0.97 | 1.22 ± 0.82 | *Taste-salty* |
| 90 ^a^ | Ich würde kleinste Verbrennungen von überall im Haus riechen. | 1.26 ± 1.08 | 1.61 ± 0.94 | Smell-danger |
| 91 ^a^ | Wenn mein Handy in meiner Tasche vibrieren würde, würde ich das schnell spüren. | 0.69 ± 0.84 | 0.81 ± 0.83 | Touch-vibration |
| 92 | Ich finde es schwierig in einer klaren Nacht einzelne Sterne zu sehen. | 0.65 ± 0.87 | 0.59 ± 0.69 | *Vision-acuity* |

## Group Comparisons including Gender Identity

The additional results reported show the descriptive and linear model statistics, as well as the results from the scree plots and Principal Components Analysis. Means and standard deviations for the SPQ-92, SPQ-35, SPQ-13, and subscales are reported for gender identities within each group.

Supplementary Table 7. Gender identity means of the translated SPQ-92, SPQ-35, SPQ-13, and subscales.

|  | | **Gender identity** | **SPQ-92** | **SPQ-35** | **SPQ-13** | **SPQ-92 vision** | **SPQ-92 hear** | **SPQ-92**  **touch** | **SPQ-92 smell** | **SPQ-92 taste** |
| --- | --- | --- | --- | --- | --- | --- | --- | --- | --- | --- |
| **Autistic group** | | Male | 115.53 (32.4) | 51.52 (19.72) | 19.27 (8.17) | 28.09 (8.55) | 27.24 (5.49) | 20.73 (7.77) | 21.12 (9.21) | 18.33 (7.25) |
|  | | Female | 82.62 (19.09) | 29,48 (10.53) | 12.90 (5.88) | 23.33 (6.33) | 23.1 (5.90) | 13.24 (4.01) | 12.19 (6.40) | 10.76 (6.21) |
| **Non-autistic group** | | Male | 127.36 (20.01) | 55.64 (11.46) | 22.18 (5.77) | 32.82 (4.22) | 32.18 (5.05) | 22.00 (5.53) | 20.42 (7.00) | 19.94 (6.14) |
|  | | Female | 114.33 (16.91) | 49.86 (11.03) | 21.19 (6.88) | 30.48 (3.67) | 27.95 (6.75) | 20.67 (4.37) | 17.48 (5.26) | 17.76 (5.51) |
|  |  | | | | | | | | | |

# Supplemental Analysis including Verbal IQ

Supplementary Table 8. Secondary analysis accounting for effects of verbal IQ. The beta coefficient, 95% confidence intervals, and *p*-values are reported as main effects for group, gender identity, and verbal IQ.

|  |  | *Coeff.* | *95% CI* | *p* |
| --- | --- | --- | --- | --- |
| **Group difference?** | SPQ-92 | 9.18 | [4.24, 14.11] | **< .001** |
|  | SPQ-35 | 5.02 | [2.07, 7.97] | **.001** |
|  | SPQ-13 | 2.41 | [0.99, 3.83] | **.001** |
| **Gender identity difference?** | SPQ-92 | -11.90 | [-16.54, -7.27] | **< .001** |
|  | SPQ-35 | -7.23 | [-9.99, -4.46] | **< .001** |
|  | SPQ-13 | -1.94 | [-3.27, -0.60] | **.005** |
| **Verbal IQ effect?** | SPQ-92 | -4.94 | [-9.98, 0.09] | .054 |
|  | SPQ-35 | -3.19 | [-6.20, -0.19] | **.038** |
|  | SPQ-13 | -1.12 | [-2.57, 0.33] | .128 |

# PCA

### Statistical Approach

Principal Components Analysis (PCA) was conducted separately per group to investigate the latent structure of the German SPQ-92. Items with low discriminatory power (> 70%) were discarded, omitting six items (four reported by Tavassoli et al. [2014] plus an additional two). Kaiser–Meyer–Olkin (KMO) criteria and Bartlett tests assessed data suitability for PCA. The KMO threshold (> 0.5) was not met for the non-autistic group but for the autistic group. The assumption of sphericity was met for both groups. Scree plots suggested two factors for the non-autistic group and one factor for the autistic group (Supplementary Figure 1). To maintain consistency across groups and in line with Tavassoli et al. (2014), two factors were used in subsequent Varimax rotation. According to Tavassoli et al. (2014), items with high loading values (> .35) for both components were omitted. Only items with high loading values (> .35) for one component and low loading values (< .35) on the other component were selected resulting in 37 items for the non-autistic group and 41 items for the autistic group. Finally, items with high factor loadings on the first component in both groups were selected (Supplementary Table 9).

### Results

PCA resulted in a 13-item version, from which eight items are included in the SPQ-35 and one assesses hyposensitivity. All sensory modalities are covered in the 13-item version: three items assess taste, four vision, three smell, two hearing, and one touch.

*Reliability and comparability*

Similar to the SPQ-92 and SPQ-35, internal consistency was good to excellent for our short SPQ-13 (autistic: α = .86; non-autistic: α = .81). Moreover, the SPQ-13 strongly correlated with the SPQ-92 and SPQ-35 for both groups (Supplementary Table 10).

*Group Comparisons*

In line with the main text, group comparisons of the SPQ-13 revealed that participants with autism reported significantly greater sensory sensitivity than non-autistic participants (Supplementary Tale 11, and females reported significantly greater sensitivity than males (Supplementary Table 6).

*AQ and SPQ-13*

The correlation between autistic traits (AQ) and sensory sensitivity (SPQ-13) was significant in the autistic group (Supplementary Table 12; Supplementary Figure 2).

### A Brief Discussion

PCA yielded a 13-item version, demonstrating good reliability. This finding was strengthened by the significant relationship between sensory sensitivity (SPQ-13) and autistic traits (AQ) in the group of individuals with autism. While the number of items is reduced from those identified by Tavassoli et al. (2014), sampling accuracy within the non-autistic group was low (i.e., KMO), most likely due to the small sample size. Nonetheless, correlational analyses demonstrated significant relationships between the SPQ-13 and SPQ-92, and with the SPQ-35, and internal consistencies were overall satisfying.

By conducting PCA, this study provides a novel methodological approach regarding the German translation. PCA yielded a 13-item version, demonstrating good reliability. While the number of items is reduced from those identified by Tavassoli et al. (2014), sampling accuracy within the non-autistic group was low (i.e., KMO), possibly due to the small sample size. Nonetheless, correlational analyses demonstrated significant relationships between the SPQ-13 and SPQ-92, and with the SPQ-35, and internal consistencies were overall satisfying.


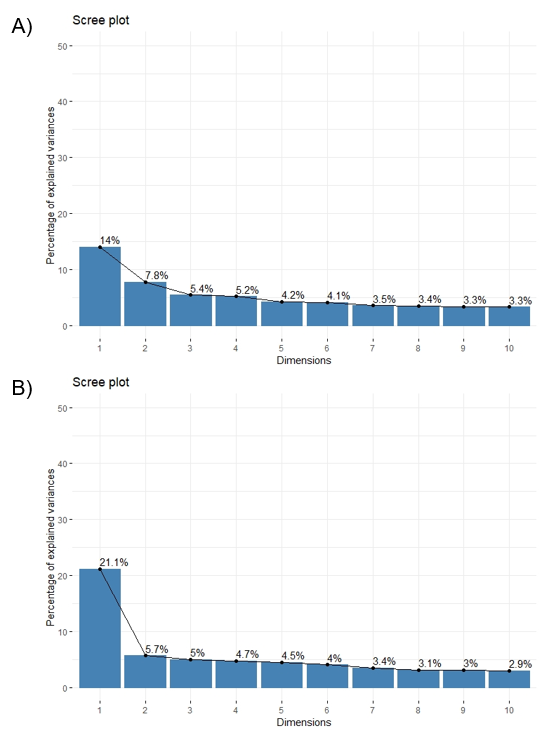


Supplementary Figure 1. Scree plots depicting the factor dimensions from the PCA of the (A) non-autistic group and (B) autistic group.

Supplementary Table 9. Itemized breakdown of the translated SPQ-92 with factor loadings from the present PCA. As reported by Tavassoli et al. (2014), the italicized items in the sensory domain were reverse scored, assessing *hyposensitivity.* ^a^ Items from the translated SPQ-35, as derived by Tavassoli et al. (2014). ^b^ Items derived from the PCA of the present study (SPQ-13).

| **Factor Loadings and item breakdown** | | **Factor Loading** | | | | **Domain** |
| --- | --- | --- | --- | --- | --- | --- |
| **Item** |  | **Autistic**  **group** | | **Non-autistic**  **group** | |  |
|  |  | Fac1 | Fac2 | Fac1 | Fac2 |  |
| 1 ^b^ | Ich würde es wahrnehmen, wenn jemand 5 Körner Salz in mein Glas Wasser zugeben würde. | 0.29 | 0.65 | 0.38 | -0.2 | Taste-salty |
| 2 ^a^ | Ich könnte verschiedene Personen anhand ihres Geruches unterscheiden. | 0.56 | 0.26 | 0.53 | -0.11 | Smell-social |
| 3 | Ich würde es nicht wahrnehmen, wenn jemand einen Löffel Zucker in meinen Tee zugeben würde. | 0.25 | 0.24 | 0.39 | 0.01 | *Taste-sweet* |
| 4 | Ich hätte keine Angst davor mir wehzutun, wenn ich bei hoher Geschwindigkeit von meinem Fahrrad fallen würde. | 0.3 | -0.37 | 0.03 | 0.02 | *Touch-pain* |
| 5 | Ich würde die Bewegung der Blätter eines rotierenden Ventilators nicht wahrnehmen sogar, wenn diese sich mit Minimalgeschwindigkeit drehen. | -0.03 | 0.34 | 0.32 | 0.46 | *Vision-motion* |
| 6 | Der Klang eines Klaviers und einer Geige, welche die gleiche Note spielen, erscheinen mir sehr ähnlich. | -0.32 | 0.01 | -0.23 | -0.33 | Hearing-complexity |
| 7 ^a^ | Ich könnte alleine anhand des Geruches erkennen, ob eine Erdbeere reif ist. | 0.6 | 0.47 | 0.25 | -0.03 | Smell-food |
| 8 | Ich könnte Milchschokolade und dunkle Schokolade alleine am Geschmack unterscheiden. | 0.61 | 0.14 | 0.37 | 0.06 | Taste-sweet |
| 9 | Ich kann heiße Duschen nicht vertragen (über 40°C / 105°F). | 0.14 | -0.24 | 0.3 | -0.14 | Touch-pain |
| 10 | Ich bräuchte keine Betäubung, um mit einer Zahnbehandlung, wie einer Zahnfüllung, klarzukommen. | 0.07 | -0.17 | -0.38 | -0.18 | *Touch-pain* |
| 11 | Ich müsste 10 Minuten warten, bis ein heißes Getränk abgekühlt ist, um es runterzuschlucken, ansonsten wäre es zu heiß für mich. | 0.3 | -0.21 | -0.03 | -0.18 | Touch-temperature |
| 12 ^a, b^ | Ich könnte visuell Änderungen in der Helligkeit einer dimmbaren Lampe erkennen, jedes Mal wenn der Dimmer um eine Stufe bewegt wird. | 0.38 | 0.33 | 0.6 | 0.03 | Vision-brightness |
| 13 | Ich könnte große Objekte, wie geparkte Autos, in einer dunklen Nacht nicht klar erkennen. | -0.11 | -0.12 | 0.22 | 0.4 | *Vision-brightness* |
| 14 ^a, b^ | Ich würde es wahrnehmen, wenn jemand 5 Tropfen Zitronensaft in mein Glas Wasser zugeben würde. | 0.19 | 0.45 | 0.59 | 0.17 | Taste-sour |
| 15 | Ich wäre die letzte Person, die bemerkt wenn etwas brennt. | 0.38 | 0.06 | 0.55 | 0.39 | *Smell-danger* |
| 16 | Ich könnte die Vibrationen lauter Musik nicht fühlen, wenn ich neben dem Lautsprecher sitzen würde (z.B. bei einem Konzert). | 0.61 | -0.08 | 0.53 | 0.35 | *Touch-vibration* |
| 17 ^b^ | Ich könnte kleine Lautstärkenveränderungen von Musik nicht als Unterschied der Vibration auf meiner Haut erkennen. | 0.02 | 0.67 | 0.59 | 0.02 | *Touch-vibration* |
| 18 | Ich kann den Fernseher nicht hören, wenn er sehr leise ist, auch wenn andere Leute es können. | 0.48 | 0 | 0.46 | 0.36 | *Hearing-loudness* |
| 19 ^a^ | Ich könnte es hören, wenn in einer ruhigen Straße ein Blatt vom Wind bewegt wird. | 0.52 | 0.35 | 0.43 | -0.1 | Hearing-loudness |
| 20 | Ich könnte nicht den Unterschied zwischen zwei Stücken dunkler Schokolade schmecken. | 0.32 | 0.44 | 0.31 | -0.12 | *Taste-sweet* |
| 21 ^a^ | Ich könnte den Unterschied zwischen zwei verschiedenen Marken salziger Kartoffelchips herausschmecken. | 0.58 | 0.61 | 0.26 | -0.03 | Taste-salty |
| 22 | Wenn Leute sprechen, scheinen die Wörter miteinander zu verschmelzen. | 0 | -0.55 | -0.04 | 0.73 | *Hearing-complexity* |
| 23 | Ich kann leuchtende Farben nur für kurze Zeit betrachten. | 0.27 | 0.53 | 0.07 | -0.65 | Vision-color |
| 24 | Ich würde sehr leicht das Gleichgewicht verlieren, wenn ich mit geschlossenen Augen auf einem Bein stehen würde. | -0.27 | -0.07 | -0.14 | -0.56 | Hearing-vestibular |
| 25 | Ich könnte einen Grill aus 20 Metern Entfernung nicht riechen. | 0.15 | 0.44 | 0.24 | 0.41 | *Smell-food* |
| 26 | Ich kann mich nicht rundherum drehen, ohne umzufallen. | 0.05 | -0.31 | -0.13 | 0.39 | *Hearing-vestibular* |
| 27 | Ich würde einen Temperaturunterschied von 10 Grad nicht bemerken. | 0.45 | 0.09 | 0.34 | 0.5 | *Touch-temperature* |
| 28 | Ich kann Tee/Kaffee schwarz trinken, ohne Milch oder Zucker zuzufügen. | 0.01 | -0.26 | -0.33 | -0.16 | *Taste-bitter* |
| 29 | Ich kann bei Musik den Bass nicht heraushören. | 0.58 | 0.11 | 0.54 | 0.41 | *Hearing-frequency* |
| 30 ^b^ | Ich könnte den Unterschied zwischen frisch geschnittenem Gras und ungeschnittenem Gras riechen. | 0.43 | 0.22 | 0.43 | 0.18 | Smell-neutral |
| 31 ^a^ | Ich könnte das Etikett auf der Rückseite meines Oberteils nicht spüren, auch wenn ich darauf achten würde. | 0.64 | 0.17 | 0.36 | 0.04 | *Touch-pressure* |
| 32 ^a, b^ | Ich kann das Summen von Elektrizität in der Wand hören. | 0.34 | 0.42 | 0.45 | -0.32 | Hearing-frequency |
| 33 ^a, b^ | Ich kann das Flackern eines Computerbildschirmes erkennen, auch wenn das Gerät ordnungsgemäß funktioniert. | 0.42 | 0.57 | 0.57 | -0.16 | Vision-motion |
| 34 | Ich könnte nicht sagen, ob Milch verdorben ist, indem ich einfach daran rieche. | 0.28 | 0.12 | 0.34 | 0.22 | *Smell-food* |
| 35 ^a^ | Ich könnte kleine Veränderungen (z.B. 1 Grad Celsius) in der Temperatur des Wetters erkennen. | 0.45 | 0.4 | -0.11 | 0.04 | Touch-temperature |
| 36 ^a^ | Ich würde einen millimetergroßen Schnitt in meiner Haut spüren. | 0.38 | -0.03 | 0.25 | -0.19 | Touch-pain |
| 37 ^b^ | Ich könnte die Bewegung einzelner Blätter eines rotierenden Ventilators sehen, selbst bei Maximalgeschwindigkeit. | 0.05 | 0.43 | 0.41 | -0.06 | Vision-motion |
| 38 ^a^ | Ich könnte mit geschlossenen Augen den Gewichtsunterschied von zwei Münzen erkennen, die auf meinen Handflächen liegen. | 0.18 | 0.73 | 0.07 | -0.32 | Touch-pressure |
| 39 | Mir würde auf einem Karussell selbst bei hoher Geschwindigkeit nicht schwindelig werden. | 0.03 | -0.29 | -0.09 | -0.07 | *Hearing-vestibular* |
| 40 | Ich kann geschrieben Wörter auf einer Seite nicht sehen, die andere Leute sehen können. | 0.5 | -0.11 | 0.55 | 0.38 | *Vision-acuity* |
| 41 | Ich könnte zwei Orangen ausschließlich am Geschmack unterscheiden. | 0.46 | 0.68 | 0.13 | -0.32 | Taste-sour |
| 42 ^a^ | Ich könnte eine bekannte Person nicht anhand ihres Geruches von einer fremden Person unterscheiden. | 0.62 | 0.28 | 0.34 | 0.07 | *Smell-social* |
| 43 ^a^ | Ich könnte nicht nur anhand des Geruches erkennen, ob ein Brot altbacken ist. | 0.01 | 0.01 | 0.15 | -0.17 | *Smell-food* |
| 44 | Ich kann nicht nur anhand des Geruches sagen, ob meine Kleidung sauber oder schmutzig ist. | 0.27 | 0.22 | 0.04 | 0.23 | *Smell-neutral* |
| 45 ^a^ | Ich könnte das Geräusch eines Staubsaugers in jedem anderen Raum eines zweistöckigen Gebäudes erkennen. | 0.45 | 0.14 | 0.58 | -0.15 | Hearing-frequency |
| 46 | Ich könnte nicht den Unterschied zwischen geradem und ungeradem Grund wahrnehmen, wenn ich auf dem Rücksitz eines Autos sitze und darüber fahre. | 0.23 | 0.1 | 0.33 | 0.22 | *Touch-vibration* |
| 47 | Ich könnte eine Tasse kochendes Wasser trinken, sofort nachdem es aus dem Wasserkocher gegossen wurde. | 0.37 | -0.52 | -0.18 | 0.41 | *Touch-pain* |
| 48 | Ich könnte nicht den Unterschied zwischen zwei grünen Äpfeln allein anhand ihrer Farbe wahrnehmen. | 0.36 | 0.08 | -0.15 | 0.21 | *Vision-color* |
| 49 ^b^ | Ich könnte ein altes und ein neues Buch anhand ihrer Gerüche unterscheiden. | 0.56 | 0.26 | 0.59 | 0.07 | Smell-neutral |
| 50 | Ich könnte ein Straßenschild aus 30 Metern Entfernung lesen. | 0.15 | -0.1 | -0.01 | 0.36 | Vision-acuity |
| 51 | Ich kann nicht sagen, ob Autos, die auf der Straße an mir vorbeifahren, mit unterschiedlicher Geschwindigkeit fahren. | 0.56 | -0.07 | 0.01 | 0.44 | *Vision-motion* |
| 52 ^b^ | Ich könnte es wahrnehmen, wenn jemand 5 Körner Zucker in mein Glas Wasser zugeben würde. | 0.27 | 0.59 | 0.41 | -0.15 | Taste-sweet |
| 53 | Ich hätte Schwierigkeiten damit, ein einzelnes Blatt klar zu sehen, sogar auf einem nahestehenden Baum. | -0.5 | 0.08 | -0.51 | -0.6 | Vision-acuity |
| 54 | Ich würde es nicht schmecken, wenn jemand einen ganzen Teelöffel Salz in mein Glas Wasser zugeben würde. | 0.37 | 0.02 | 0.16 | 0.06 | *Taste-salty* |
| 55 ^a^ | Ich könnte den elastischen Bund meiner Socken spüren, wenn ich stehen bleiben und darauf achten würde. | 0.39 | 0.43 | 0.16 | -0.5 | Touch-pressure |
| 56 | Ich kann nicht den Unterschied zwischen reifen und unreifen Früchten schmecken. | 0.51 | -0.12 | 0.33 | 0.21 | *Taste-sweet* |
| 57 | Ich könnte für fünfzehn Sekunden auf einem Bein stehen ohne zu wackeln. | -0.06 | 0.19 | -0.29 | -0.3 | *Hearing-vestibular* |
| 58 ^a^ | Ich könnte den Unterschied zwischen augenscheinlich gleichen Süßigkeiten herausschmecken. | 0.35 | 0.5 | -0.02 | -0.19 | Taste-sweet |
| 59 ^a^ | Ich nehme das Gewicht und den Druck eines Hutes auf meinem Kopf wahr. | 0.57 | 0 | 0.43 | -0.1 | Touch-pressure |
| 60 ^a^ | Ich würde die Berührung eines einzelnen Haares auf meinem Handrücken spüren. | 0.47 | -0.02 | 0.59 | -0.08 | Touch-pressure |
| 61 ^a^ | Wenn ich entlanggehe, würde ich selbst mit geschlossenen Augen die Vibrationen eines vorbeifahrenden Lasters spüren. | 0.6 | 0.44 | 0.42 | 0.12 | Touch-vibration |
| 62 ^a^ | Ich könnte den kleinsten Gasaustritt von überall im Haus riechen. | 0.58 | 0.5 | 0.33 | -0.09 | Smell-danger |
| 63 ^a^ | Ich würde nicht anhand des Geruches erkennen, falls jemand das Parfum gewechselt hätte. | 0.3 | 0.31 | 0.49 | 0.07 | *Smell-social* |
| 64 | Ich könnte sagen, wenn ein Fahrstuhl sich in Bewegung setzen würde. | 0.21 | -0.11 | 0.44 | 0.47 | Hearing-vestibular |
| 65 | Ich kann sehr leicht Hundepfeifen im Park hören. | 0.07 | 0.35 | 0.17 | 0.05 | Hearing-frequency |
| 66 | Ich würde nicht den Unterschied zwischen verschiedenen Sorten von Salatblättern schmecken. | 0.66 | 0.33 | 0.51 | 0.02 | *Taste-bitter* |
| 67 | Ich könnte nicht schmecken, falls in meinem Wasserglas zwei Scheiben Zitrone wären, wenn ich es mit geschlossenen Augen trinken würde. | 0.36 | 0.02 | 0.52 | 0.39 | *Taste-sour* |
| 68 ^a^ | Ich kann bei hellem Sonnenschein nicht ohne Sonnenbrille rausgehen. | 0.41 | 0.15 | 0.04 | -0.46 | Vision-brightness |
| 69 | Ich könnte Kleingedrucktes, wie eine Seriennummer auf der Rückseite einer DVD, aus 3 Metern Entfernung lesen. | 0.04 | 0.12 | 0.29 | -0.28 | Vision-acuity |
| 70 | Ich bekomme leicht Reiseübelkeit (z.B. im Auto oder Seekrankheit) | 0.24 | 0.14 | -0.34 | -0.08 | Vision-motion |
| 71 ^a^ | Ich könnte den Temperaturunterschied einer Tasse frischen Kaffees spüren, wenn dieser eine Minute herumstand. | 0.46 | 0.55 | 0.46 | -0.16 | Touch-temperature |
| 72 | Ich kann sehr tiefe Töne, wie tiefe Stimmen, nicht hören. | 0.28 | -0.32 | 0.53 | 0.45 | *Hearing-frequency* |
| 73 ^a, b^ | Ich würde es als Erste/r hören, wenn eine Fliege im Raum ist. | 0.31 | 0.43 | 0.41 | 0.06 | Heairng-loudness |
| 74 ^a^ | Wenn ich einen Stapel blauer Pullover im Laden sehe, die identisch sein sollen, könnte ich Unterschiede zwischen ihnen erkennen. | 0.26 | 0.56 | 0.32 | -0.43 | Vision-color |
| 75 ^a^ | Ich würde einen neuen Geruch in meinem Zuhause nicht direkt vor allen anderen bemerken. | 0.53 | 0.19 | 0.45 | 0.04 | *Smell-neutral* |
| 76 | Ich besitze absolutes Gehör: z.B. könnte ich eine Musiknote ohne Hilfe wiederholen. | 0.11 | 0.45 | 0.19 | -0.34 | Hearing-complexity |
| 77 | Ich könnte ohne irgendwelche Probleme in eine Zitrone beißen. | 0.04 | -0.17 | -0.42 | -0.05 | *Taste-sour* |
| 78 | Ich bräuchte im Winter keinen Mantel zu tragen, selbst wenn es null Grad draußen ist. | 0.03 | -0.36 | 0.16 | 0.17 | *Touch-temperature* |
| 79 | Ich könnte nicht die Farbe eines Pullovers im Laden an die Farbe meiner Hose zuhause anpassen. | 0.44 | 0.04 | 0.05 | 0.31 | *Vision-color* |
| 80 | Ich würde nicht jede einzelne Note hören, wenn ich Musik höre. | 0.39 | 0.21 | 0.11 | -0.2 | *Hearing-frequency* |
| 81 ^a, b^ | Ich könnte den Unterschied zwischen den meisten Männern und Frauen riechen. | 0.23 | 0.49 | 0.68 | -0.15 | Smell-social |
| 82 | Ich entscheide mich dazu, gedeckte Farben zu tragen. | 0.15 | 0.19 | 0.27 | -0.29 | Vision-brightness |
| 83 | Ich höre Musik bei minimaler Lautstärke. | 0.24 | 0.11 | 0.01 | -0.22 | Hearing-loudness |
| 84 ^a^ | Ich könnte jede Note eines Akkords hören, auch wenn es 10 Noten wären. | 0.16 | 0.4 | 0.29 | -0.34 | Hearing-complexity |
| 85 ^a^ | Ich schließe Vorhänge, um helles Licht zu vermeiden. | 0.41 | 0.23 | -0.16 | -0.59 | Vision-acuity |
| 86 | Ich könnte nicht den Unterschied in einem Ton hören, wenn das gleiche Instrument die gleiche Note zu unterschiedlichen Zeiten spielt. | 0.13 | 0.36 | 0.02 | 0.12 | *Hearing-complexity* |
| 87 ^a, b^ | Ich könnte zwei verschiedene Sorten Kaffee anhand ihres Geruches unterscheiden, sogar mit geschlossenen Augen. | 0.51 | 0.66 | 0.51 | -0.32 | Smell-food |
| 88 ^a, b^ | Ich kann in den meisten Umgebungen Staubpartikel in der Luft sehen. | 0.29 | 0.67 | 0.51 | -0.05 | *Vision-acuity* |
| 89 ^a^ | Ich könnte den Unterschied zweier Tomatensoßen mit unterschiedlichem Salzgehalt nicht herausschmecken. | 0.56 | 0.4 | 0.45 | 0.07 | Taste-salty |
| 90 ^a^ | Ich würde kleinste Verbrennungen von überall im Haus riechen. | 0.52 | 0.4 | 0.47 | 0 | Smell-danger |
| 91 ^a^ | Wenn mein Handy in meiner Tasche vibrieren würde, würde ich das schnell spüren. | 0.18 | 0.43 | 0.27 | 0.44 | Touch-vibration |
| 92 | Ich finde es schwierig in einer klaren Nacht einzelne Sterne zu sehen. | 0.42 | 0.12 | 0.12 | 0.39 | *Vision-acuity* |

Supplementary Table 11. Group comparison of the derived SPQ-13. Means and standard deviations for the SPQ-13 are reported for each group. The beta coefficient (*B*) and 95% Confidence Intervals (*CI*) are reported as main effects, with significance levels of 0.05 indicated (** *p* < .01, *** *p* < .001).

|  |  | | **SPQ-13**  Mean (SD) |
| --- | --- | --- | --- |
| **Autistic group** |  | | 16.80 (7.95) |
|  |  |  |  |
| **Non-autistic group** |  | | 21.80 (6.18) |
|  |  |  |  |
| **Group difference?** | *Β* | | 2.80*** |
|  | *95% CI* | | [1.46, 4.13] |
| **Gender difference?** | *Β* | | -1.85** |
|  | *95% CI* | | [-3.18, -0.50] |

Supplementary Table 12. Correlation matrix of sensory sensitivity, autistic traits, and verbal IQ. Pearson’s *r* correlation coefficients. Bolded values remained significant (*p* < .05) after Bonferroni correction.

|  | **SPQ-92** | **SPQ-35** | **SPQ-13** | **AQ** |
| --- | --- | --- | --- | --- |
| **Autistic group** |  |  |  |  |
| SPQ-92 | - |  |  |  |
| SPQ-35 | **0.96** | - |  |  |
| SPQ-13 | **0.84** | **0.88** | - |  |
| AQ | -0.37 | -0.41 | **-0.46** | - |
| WST | -0.08 | -0.05 | -0.12 | 0.14 |
| **Non-autistic group** |  |  |  |  |
| SPQ-92 | - |  |  |  |
| SPQ-35 | **0.88** | - |  |  |
| SPQ-13 | **0.77** | **0.81** | - |  |
| AQ | 0.00 | -0.09 | -0.09 | - |
| WST | -0.10 | -0.18 | -0.01 | -0.05 |


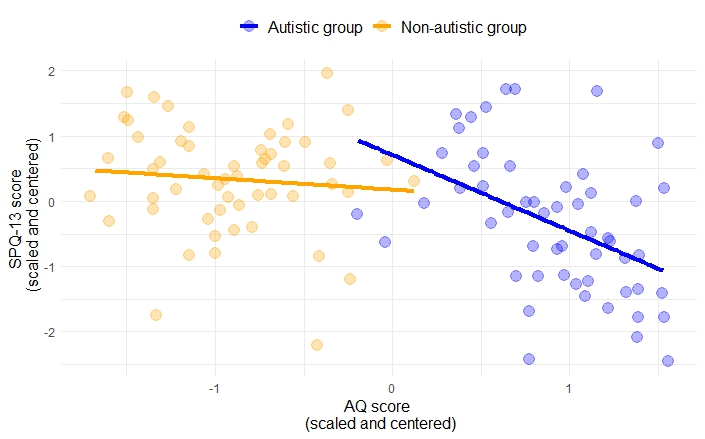


Supplementary Figure 2. Data from the derived SPQ-13 and the AQ for both groups.

# Supplementary Analysis with AQ cutoff criteria applied

Similar to the exclusion criteria used by Tavassoli et al. (2014), autistic participants that scored below 27 on the AQ and non-autistic participants with an AQ score above 27 were excluded. This resulted in the exclusion of two non-autistic participants (trimmed non-autistic group: Gender identity: 20 female, 32 male; age: *M* = 42.87 ± 12.94 [range: 24 - 62 years]; verbal IQ: *M* = 96.5 ± 7.87).

Supplementary Table 13. Group comparison of age and verbal IQ with trimmed non-autistic group (AQ < 27). Data were non-normally distributed but varied homogeneously for age and verbal IQ. Thus, Mann-Whitney *U* tests were conducted to compare the groups. The groups did not differ by age but did by verbal IQ.

|  | **Normality** | | **Homogeneity of Variance** | | **Mann-Whitney *U*** | |
| --- | --- | --- | --- | --- | --- | --- |
|  | W | *p* | Levene’s | *p* | *U* | *p* |
| **age** | 0.91 | **<.001** | 0.42 | .52 | 1351 | 0.74 |
| **verbal IQ** | 0.95 | **<.001** | 3.09 | .08 | 698 | **<.001** |

Supplementary Table 14. Correlation matrix of sensory sensitivity, autistic traits, and verbal IQ with trimmed non-autistic group (AQ < 27). Given that only the non-autistic group changed, the group with autism is reported for completeness. After Bonferroni correction, all SPQ versions were highly correlated in both groups. The SPQ-13 also strongly correlated with autistic traits (AQ).

|  | **SPQ-92** | **SPQ-35** | **SPQ-13** | **AQ** |
| --- | --- | --- | --- | --- |
| **Autistic group** |  |  |  |  |
| SPQ-92 |  |  |  |  |
| SPQ-35 | **0.96** |  |  |  |
| SPQ-13 | **0.84** | **0.88** |  |  |
| AQ | -0.37 | -0.41 | **-0.46** |  |
| WST | -0.09 | -0.04 | -0.13 | 0.16 |
| **Non-autistic group** |  |  |  |  |
| SPQ-92 |  |  |  |  |
| SPQ-35 | **0.88** |  |  |  |
| SPQ-13 | **0.78** | **0.81** |  |  |
| AQ | 0.11 | -0.08 | -0.12 |  |
| WST | -0.07 | -0.16 | -0.02 | -0.16 |

Supplementary Table 15. Group comparison of the translated SPQ-92, SPQ-35, and SPQ-13 with trimmed non-autistic group (AQ < 27). Given that only the non-autistic group changed, the group with autism is reported for completeness. Mean and standard deviation scores for the SPQ-92, SPQ-35, and SPQ-13 are reported for gender within each group. The beta coefficient, 95% confidence intervals, and *p*-values are reported as main effects.

|  |  | | **SPQ-92** | **SPQ-35** | **SPQ-13** |
| --- | --- | --- | --- | --- | --- |
| **Autistic group** |  | | 102.72 (32.14) | 42.94 (19.86) | 16.80 (7.95) |
|  |  |  |  |  |  |
| **Non-autistic group** |  | | 122.42 (20.02) | 52.52 (11.74) | 21.75 (6.3) |
|  |  |  |  |  |  |
|  |  |  |  |  |  |
| **Group difference?** | *Β* | | 10.95 | 6.18 | 2.77 |
|  | *95% CI* | | [6.18, 15.72] | [3.32, 9.03] | [1.40, 4.13] |
|  | *p* | | **< .001** | **< .001** | **< .001** |
| **Gender difference?** | *Β* | | -11.38 | -6.96 | -1.88 |
|  | *95% CI* | | [-16.15, -6.61] | [-9.82, -4.10] | [-3.24, -0.51] |
|  | *p* | | **< .001** | **< .001** | **.007** |

**Supplementary Analysis Summary:** The results of the supplementary analysis mirror that of the reported analysis, whereby the same outcomes for group comparison of age and verbal IQ, as well as the correlation analyses within the groups, produced the same results. Moreover, the group comparisons also demonstrated the same outcomes as the reported sample. Thus, we can infer that including the two non-autistic participants with AQ scores exceeding 27 was not influential in the overall interpretation of the reported analysis in the main manuscript.
